# Neural oscillations arising from a linear current with negative conductance

**DOI:** 10.1186/1471-2202-14-S1-P32

**Published:** 2013-07-08

**Authors:** Farzan Nadim, Yinzheng Guan, Jorge Golowasch, Amitabha Bose

**Affiliations:** 1 Department of Biological Sciences, NJIT-Rutgers University, Newark, NJ 07102, USA; 2 Department of Mathematical Sciences, NJIT, Newark, NJ 07102, USA

## Introduction

Slow neural oscillations are known to depend on regenerative inward currents. The voltage range in which these currents dominate the neuron's activity is typically limited to the negative-slope conductance range of their IV curve. At low voltages, these currents are not active and at high voltages, outward currents and spike-activity dominate. We find that the contribution of such currents to neural oscillations can be approximated by a linear current with negative slope conductance (*I_NL_*). Here we explore the minimal set of requirements to produce neural oscillations in the presence of *I_NL _*with the goal of elucidating how other current types shape the properties of oscillations.

## Methods

The pacemaker PD neurons in the crab pyloric network are quiescent in the absence of modulatory inputs. We have previously shown that adding *I_NL _*with dynamic clamp restores oscillatory activity in these neurons. We now examine whether dynamic clamp *I_NL _*can recover oscillations in the absence of spiking (in TTX). We explore the generation of oscillations with *I_NL _*using phase-space and bifurcation analysis of simple mathematical models.

## Results

We find that adding *I_NL _*in the presence of TTX is sufficient to produce oscillatory activity in PD neurons (Fig. A). To understand how oscillations can be generated by adding this simple linear current, we analyzed a 2-variable conductance-based model which includes *I_NL _*and an outward current *I_K _*with a single activation variable. We find that this model is able to reproduce the PD oscillations but these oscillations are limited to a small range of *g_NL _*values (Fig. B).

**Figure 1:**
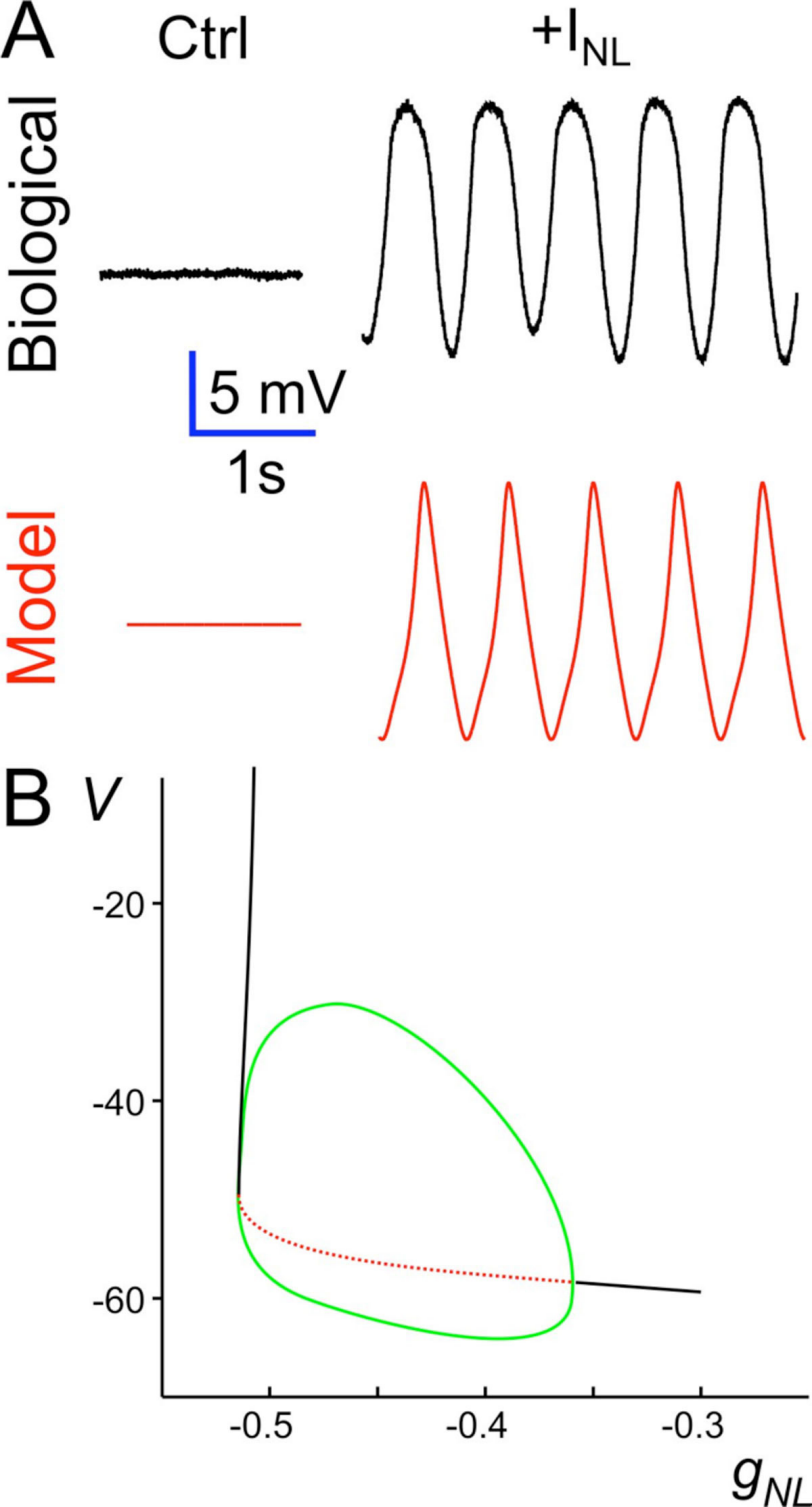
**A. Addition of *I_NL _*to a biological PD neuron in TTX results in oscillatory activity, an effect mimicked by a 2d model containing *I_NL _*and *I_K_***. (Ctrl: -50 mV.) **B**. The 2d model bifurcation diagram with *g_NL _*as parameter shows a limited range of oscillatory activity bound by two Hopf bifurcation points.

The dynamics of a quiescent neuron are locally represented by a stable equilibrium point. Adding *I_NL _*destabilizes this equilibrium point. In the absence of a limiting factor, the voltage would grow without bounds. At depolarized voltages, this limiting factor is provided by *I_K_*. Surprisingly, the presence of *I_K _*together with *I_NL _*also limits the voltage from escaping in the negative direction and thus results in oscillatory activity (Figure 1B). These oscillations require a delicate balance between *I_K _*and *I_NL_*, leading to restrictions in parameter space where rhythmic activity exists. We show that oscillations are readily stabilized in the model by adding a hyperpolarization-activated current such as *I_h_*.

## Conclusions

The existence of *I_NL _*is sufficient to destabilize a stable equilibrium point around which oscillations can be produced. However, *I_NL _*must interact with other membrane currents to be able to bind? the voltage both at hyperpolarized and depolarized levels. The existence of these bounds depends on the intrinsic properties of individual neurons which may determine whether or not a neuron can oscillate using *I_NL_*. Thus, these results suggest that adding *I_NL _*to other pyloric neurons may not lead to oscillations and whether it does will depend on what other ionic currents they express. Our current work involves identifying which pyloric neurons respond to *I_NL _*with oscillatory activity and which not, and how that depends on the intrinsic properties of these cells.
